# Kinetic Trapping of Charge-Transfer Molecules at Metal Interfaces

**DOI:** 10.1021/acs.jpcc.3c08262

**Published:** 2024-02-07

**Authors:** Anna Werkovits, Simon B. Hollweger, Max Niederreiter, Thomas Risse, Johannes J. Cartus, Martin Sterrer, Sebastian Matera, Oliver T. Hofmann

**Affiliations:** † Institute of Solid State Physics, Graz University of Technology, Petersgasse 16/II, 8010 Graz, Austria; ‡ Institute of Physics, University of Graz, Universitätsplatz 5, 8010 Graz, Austria; § Institut für Chemie und Biochemie, Freie Universität Berlin, Arminallee 22, 14195 Berlin, Germany; ∥ Theory Department, Fritz Haber Institute of the MPG, Faradayweg 4-6, 14195 Berlin-Dahlem, Germany

## Abstract

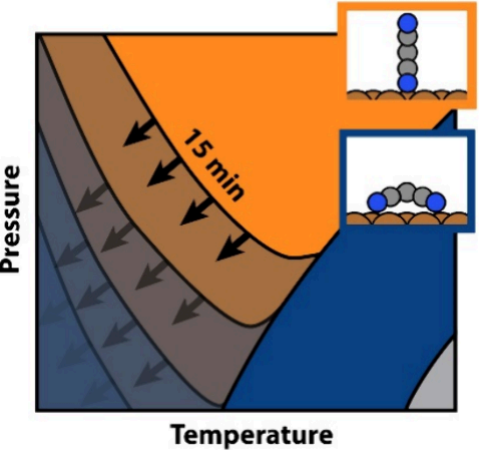

Despite the common expectation that conjugated organic molecules on metals adsorb in a flat-lying layer, several recent studies have found coverage-dependent transitions to upright-standing phases, which exhibit notably different physical properties. In this work, we argue that from an energetic perspective, thermodynamically stable upright-standing phases may be more common than hitherto thought. However, for kinetic reasons, this phase may often not be observed experimentally. Using first-principles kinetic Monte Carlo simulations, we find that the structure with lower molecular density is (almost) always formed first, reminiscent of Ostwald’s rule of stages. The phase transitions to the upright-standing phase are likely to be kinetically hindered under the conditions typically used in surface science. The simulation results are experimentally confirmed for the adsorption of tetracyanoethylene on Cu(111) using infrared and X-ray photoemission spectroscopy. Investigating both the role of the growth conditions and the energetics of the interface, we find that the time for the phase transition is determined mostly by the deposition rate and, thus, is mostly independent of the nature of the molecule.

## Introduction

The extensive polymorphism exhibited by inorganic/organic interfaces can be both a blessing and a curse, as many properties, such as the interface dipole^1^ or the charge-carrier mobilities^2^ are strongly affected by the structure at the interface.^3^ A prototypical example of this is found in lying-to-standing phase transitions. These occur, e.g., when at low dosages molecules assume a flat-lying structure, but upon deposition of more material, the first layer reorients into a more tightly packed, upright-standing structure. Such structural changes are often accompanied by a sudden, large change of the molecules’ electron affinity^1^ and, consequently, the interface dipole.^4^

Generally, upright-standing phases quickly form when intralayer interactions dominate over adsorbate–substrate interactions, which is commonly the case for adsorption of conjugated molecules on semiconducting organic^5,6^ or inorganic^7,8^ substrates. Also on metallic substrates, lying-to-standing phase transitions are found for covalently bonded self-assembled monolayers,^9−11^ i.e., organic molecules that interact weakly with the surface (e.g., mostly through van der Waals interactions and no or only a single docking group) and where intralayer interactions dominate. Conversely, for conjugated organic molecules that have multiple functional groups and which undergo charge-transfer reactions with the surface, it appears that most systems^12−28^ lack indications for upright-standing structures – a few notable exceptions notwithstanding.^29−31^

Although it is conceivable that in some cases, the reoriented (standing) phase never becomes thermodynamically stable, the absence of experimental evidence for these structures is not sufficient to conclude their thermodynamic instability. From a thermodynamic point of view, such lying-to-standing phase transitions should be very common also for molecules with strong molecule–substrate interactions (see the Supporting Information for a detailed discussion). A possible explanation that has hitherto not received much attention would be that the phase transition is kinetically prevented.

Experimentally, kinetic trapping is extremely difficult to address. This prompted us to conduct a joint study using growth experiments and first-principles kinetic Monte Carlo (kMC) simulations to investigate (1) under which growth conditions kinetically trapped phases are likely to occur, (2) how long a kinetically trapped phase would be expected to be stable before it transitions to the thermodynamic minimum, and (3) how the formation of a kinetically trapped phase depends on the nature of the organic adsorbate. These simulations demonstrate that in conditions commonly used in surface science, the flat-lying phase becomes kinetically trapped (almost) independently of material-dependent parameters, such as the adsorption energies or the barriers for diffusion. We verify this prediction exemplarily for the deposition of tetracyanoethylene (TCNE) on Cu(111) using X-ray photoemission and infrared reflection absorption spectroscopy.

**Figure 1:**
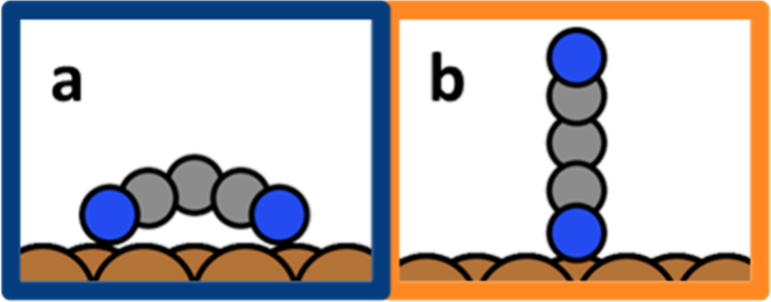
(a) Flat-lying TCNE and (b) upright-standing TCNE on Cu(111).

To keep the following discussion simple and general, we employ several approximations of the simulation of the growth process. As a first approximation, we focus only on the layer in direct contact with the surface, neglecting adsorption in the second layer or beyond. As second approximation, we neglect intermolecular interactions altogether, since here we want to conceptually study the situation where intermolecular interactions do not constitute a driving force toward upright-standing layers. Finally, for our simulations, we stipulate that the molecule can only adsorb in two states, flat-lying or upright-standing, as shown in Figure 1. Reality is more complicated (there are different possible sites with slightly different energies),^31^ but the restriction to two states simplifies the discussion here without changing the qualitative picture.

Generally speaking, in thermodynamic equilibrium the most stable structure is the one that minimizes Gibbs energy per area γ^32^:
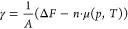
1where *A* is the area of the unit cell, Δ*F* is the free energy of the structure, μ is the chemical potential of the molecular reservoir depending on its pressure and temperature, and *n* is the number of molecules per unit cell. The competition between the two polymorphs occurs because conjugated organic molecules can pack more densely in an upright-standing adsorption geometry, but the adsorption energy is larger (more exergonic) when they maximize the contact area with the substrate, i.e., adsorb flat-lying. Consequently, the flat-lying polymorph is thermodynamically stable at low pressures and high temperatures, while the upright-standing polymorph is thermodynamically preferred at high pressures and low temperatures.

## Methods

### Computational Details

We model the microkinetic behavior by kinetic Monte Carlo (kMC) simulations via the *kmcos* code.^33^ The ingredients required are the molecular adsorption sites and their kinetic interconnections. The latter are manifested through the elementary processes our prototypical molecules undergo in a PVD experiment, i.e., adsorption/desorption, diffusion, and reorientation. In detail, elementary processes are quantified spatially through their initial and final adsorption sites, as well as, temporally through their process rates. According to the Variable Step Size Method,^34−36^ at each step, one process is randomly drawn (processes weighted by their process rates) and executed after the simulation time is forwarded by a random number distributed according to the Poisson statistic of the total rate of processes possible at the kMC step. Initially, we start with an empty surface, on which molecules collide with the surface with a given impingement rate, given as^36^
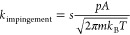
2where *p* is the pressure, *m* is the mass of the molecule, and *A* is the area. We note in passing that flat-lying molecules occupy twice the area of a standing molecule. The sticking coefficient *s* is set to unity here, but adsorption is only permitted if the adjacent unit cells are still unoccupied, i.e., if there is space for the molecule to adsorb in its respective geometry. Once a molecule is adsorbed on the surface, it is free to diffuse and rotate or to desorb. Furthermore, upright-standing molecules can fall over, or lying molecules can stand up. The rate constants *k* of these processes are modeled using the Arrhenius equation
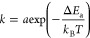
3where Δ*E*_a_ is the activation energy, *a* is the attempt frequency, *k*_B_ is the Boltzmann constant, and *T* is the temperature. Naturally, desorption is the slowest process because the (negative) adsorption energy is much higher than the barriers for all other processes and the pre-exponential factors are similarly large (Supporting Information). Conversely, diffusion and rotation on the surface exhibit not too large barriers and are relatively fast. The most critical rates, however, are the reorientation processes. Because of the significantly more stable flat-lying adsorption geometry, “falling over” is a much faster process than “standing up” (see ref (43)). Details about the approximations as well as the used transition rate constants are stated in the Supporting Information.

To circumvent the common time disparity problem, we applied the time acceleration method by Dybeck et al.^38,39^ As discussed above, intermolecular interactions are neglected. All simulations are conducted on a lattice with 20 × 20 sites, starting with an empty surface. To incorporate statistic effects, the simulations at one distinct (*p,T*)-point are repeated are repeated 5 times with different random seeds. We sampled (*p,T*)-points with temperature steps of 10 K and pressure steps of 2 powers of ten mbar.

**Figure 2:**
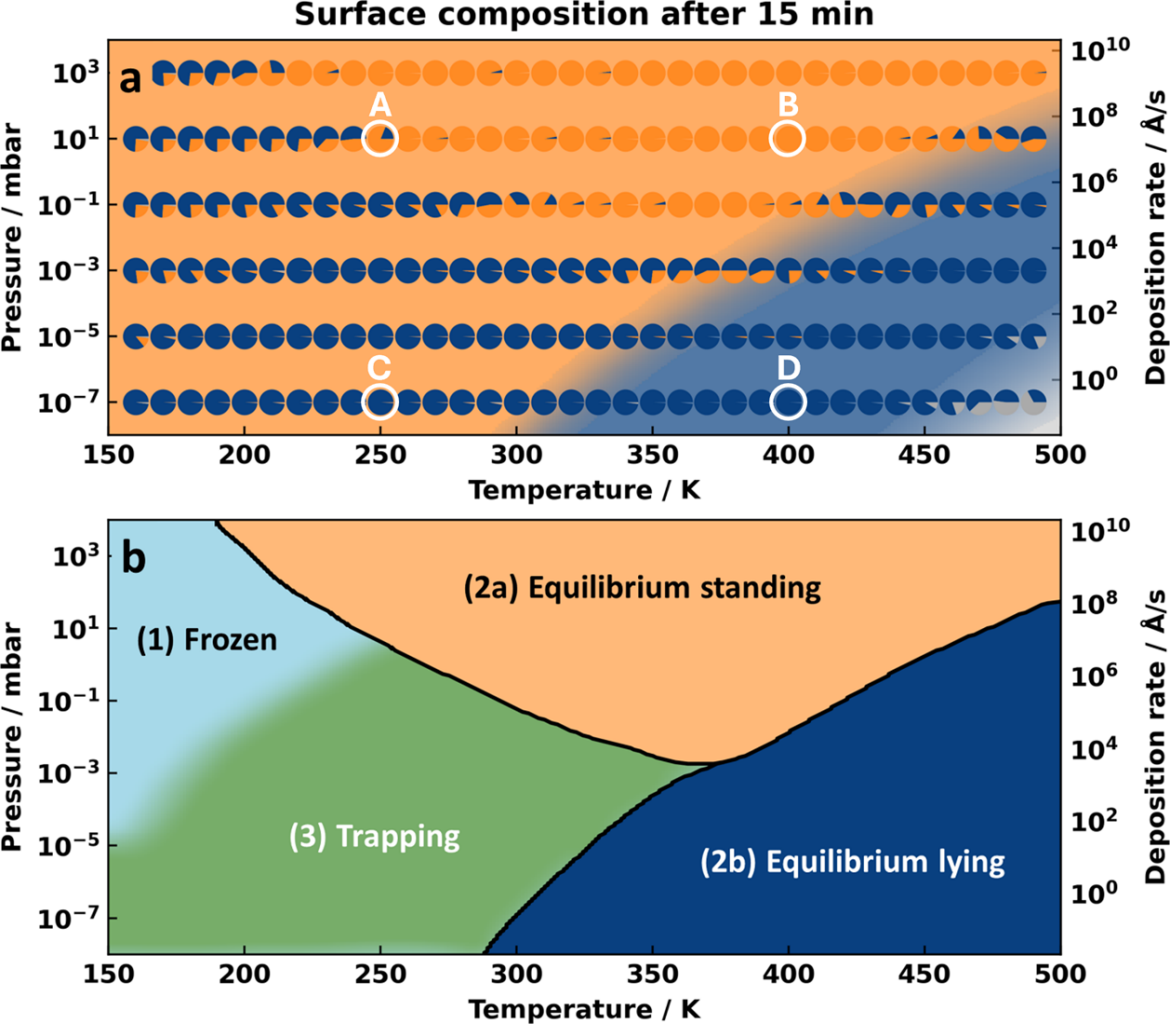
(a) Thermodynamic phase diagram according to eq 1 is shown in the background. In the foreground, the pie-charts represent the relative composition of the surface after 15 min have passed at the same conditions. Blue denotes the percentage of the area covered with lying molecules, orange the percentage of area covered with standing molecules, and gray empty surface. For the conditions highlighted with a white circle, (A–D), the time evolution of the surface composition as a function of time is shown in Figure 4. (b) Qualitative assignment of the diagram into different regions (discussion see main text).

The conversion from pressure to the deposition rate *r* in Å/s as indicated on the secondary axis of Figure 2 is
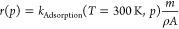
4where *k*_Adsorption_ is the adsorption rate from eq 1, *m* is the mass of the molecule, and ρ is the mass density of the molecule in the bulk. We keep the temperature constant at 300 K since the deposition rate only varies slowly with the inverse square root of the temperature.

### Experimental Details

The setup used for the experiments consists of a UHV chamber system equipped with a low energy electron diffraction (LEED) apparatus, a dual-anode (Al, Mg) X-ray source and a hemispherical electron analyzer for X-ray photoelectron spectroscopy (XPS) and an attached Bruker Vertex 80v FTIR spectrometer with an external mercury–cadmium-telluride (MCT) detector for infrared reflection absorption (IRRAS) spectroscopy. The Cu(111) single crystal was cleaned by repeated cycles of Ar^+^ ion bombardment and annealing at 850 K until a clean and well-ordered surface was obtained, as checked with LEED and XPS. TCNE was dosed from an evacuated glass vessel through a leak valve. Prior to the dosing experiments, TCNE was resublimated to increase its purity. The XPS measurements were performed at normal emission. A previously reported XPS fitting procedure has been adapted to obtain the distribution of flat-lying and upright-standing TCNE molecules in the monolayer from the C 1s and N 1s spectra.^37,40^ For IRRAS measurements, the resolution was set to 4 cm^–1^ and between 100 and 500 scans were accumulated for one spectrum. The highest TCNE monolayer coverage, as determined from the C 1s and N 1s XPS signal intensities, was obtained by first adsorbing a multilayer of TCNE at 200 K followed by warming the sample to room temperature. This coverage is referred to as 1 ML (3.25 TCNE/nm^2^).^40^

## Results and Discussion

By definition, when running kMC simulations until equilibrium is obtained (see the Method section for details), the resulting surface composition is independent of any kinetic barriers and depends only on the relative adsorption energies Δ*F* and surface footprints *A* of standing and lying molecules. In the background of Figure 2, we exemplarily show the obtained composition diagram for an adsorption energy of 2.40 eV and a footprint of 2 molecules/nm^2^ for the flat-lying phase and an adsorption energy of 1.86 eV and a footprint of 4 molecules/nm^2^ for the upright-standing phase. To emphasize the generality, the results for different adsorption energies are also showcased later in this work. For the sake of discussion, we include vapor pressures that are much higher than in most experiments (up to 1 bar), which would correspond to deposition rates of about 1 mm per second (see Method section for conversion). The composition in equilibrium consists of a region with predominantly lying molecules (blue) and predominantly standing molecules (orange). In the following, we denote these as “phases” although there is no sharp transition line between those, even in equilibrium.^41,42^

To contrast the thermodynamic expectation with the expected results of a realistic growth experiment, we stop the kMC simulations after exposing the initially empty surface to the molecule reservoir for 15 min. The relative composition of the interface is depicted as a pie chart in the foreground of Figure 2a. To make our simulations compatible with experiments (see below), we choose barriers for diffusion and reorientation that were obtained in an earlier work^43^ for TCNE on Cu(111). Results for deviating barriers and adsorption energies are shown and discussed later in this work. Qualitatively, we can separate the graph into three different regions: (1) At very low temperatures and higher pressures, the surface shows a mixture of standing and lying molecules as all processes leading to changes in the orientation (including desorption) are essentially frozen. Here, the surface composition is simply determined by the way in which molecules adsorb on the surface. We assume that a typical planar, conjugated organic molecule is twice as likely to adsorb upright-standing (on an edge) than flat-lying (adsorbing on its face), as discussed in more detail in the Supporting Information.

This region is of no further relevance to the discussion.

In region (2), which is at high pressures or high temperatures, the outcome of the kMC simulation matches the expectation from the thermodynamic equilibrium. For the sake of clarity, we distinguish (2a), where the standing phase forms, from (2b), where the flat-lying phase forms. Finally, in region (3), we find that after a growth process of 15 min, the interface consists almost entirely of flat-lying molecules, despite the thermodynamic preference of upright-standing molecules. In these conditions, the flat-lying phase is kinetically trapped. Interestingly, here, temperature alone does not seem to be the major factor, because at similar temperatures but higher pressures/deposition rates, the stable standing phase is readily formed.

**Figure 3:**
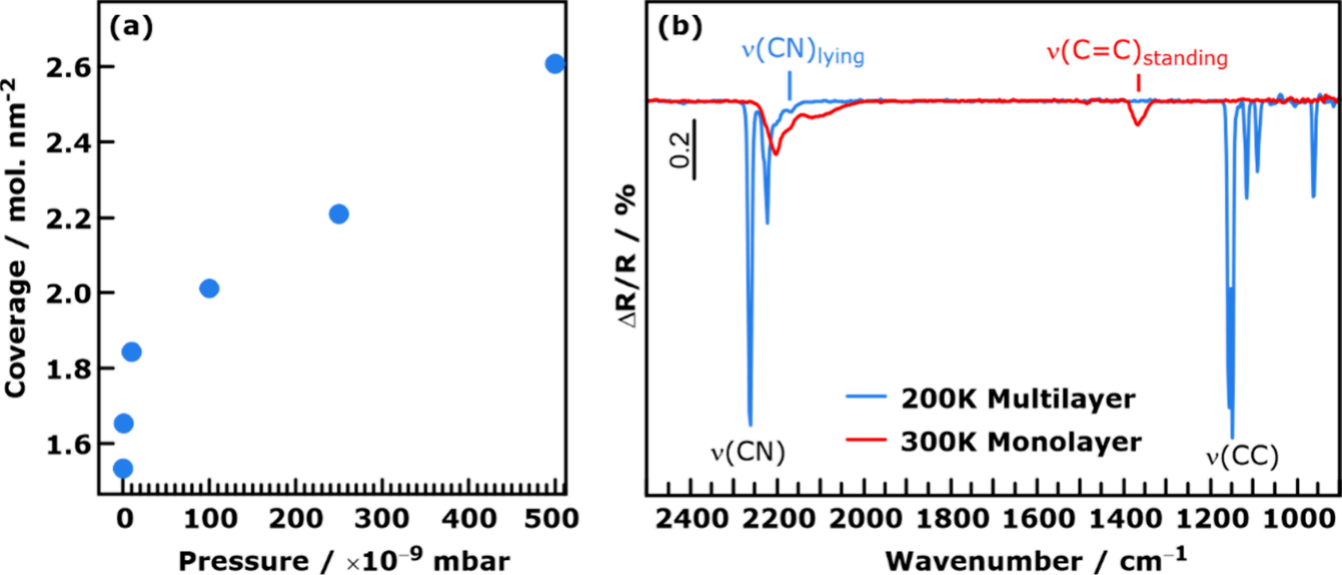
(a) Experimentally determined TCNE equilibrium coverage at 300 K. (b) Infrared reflection absorption spectra for TCNE on Cu(111) after deposition of 10 Langmuir TCNE at 200 K (blue) and subsequent heating to room temperature (red).

To confirm these computational expectations, we performed two sets of growth experiments. As indicated above, we employ TCNE on Cu(111) as exemplary system because there are strong indications of a flat-lying to upright-standing phase transition both experimentally and in theory.^31,44^ In the first experiment, we monitor the coverage of TCNE on Cu(111) using X-ray photoelectron spectroscopy (XPS), which exhibits characteristic C 1s and N 1s signals for upright-standing and flat-lying TCNE^37^ (for details about the coverage determination, see Method section). We start from a clean Cu(111) surface at 300 K and expose it to different vapor pressures of TCNE until saturation under these conditions is reached. As shown in Figure 3a, at the lowest dosing pressure considered (2 × 10^–10^ mbar), we obtain a coverage of approximately 1.6 TCNE/nm^2^, which increases quickly to 1.85 TCNE/nm^2^ at a pressure of 10^–8^ mbar, and then with a smaller slope at higher dosing pressures. Notably, the coverage measured at the lowest pressure is slightly above the 1.4 TCNE/nm^2^ for the closure of a full monolayer reported by Erley and Ibach^45^ and slightly below the 2.0 TCNE/nm^2^ we expect for a perfectly well-ordered monolayer of flat-lying molecules only.^31^ While we cannot rule out completely that initially, a low-coverage phase forms that we have not considered so far (e.g., by incorporating adatoms or because of strong repulsive interactions between the molecules), our XPS analysis suggests that under these low-pressure conditions the surface is covered with disordered molecular islands consisting predominantly of flat-lying molecules with some standing molecules, and empty space in between (areal contribution of lying:standing:empty = 5:3:2); see Figure S8 and ref (40). for more details about XP spectra fitting).^40^ At higher pressures, the remaining holes in the layer are filled with upright-standing TCNE molecules. This is qualitatively consistent with the computed “thermodynamic” phase diagram, which predicts mixed compositions at room temperature and moderate to low pressures, which is in agreement with the analysis of the composition at room temperature provided elsewhere.^40^

In a second experiment, we used infrared reflection absorption spectroscopy (IRRAS) to monitor thermally induced morphological transitions within an adsorbed TCNE monolayer. For this, TCNE was initially adsorbed on the Cu(111) surface at a temperature of 200 K, which is below the multilayer desorption temperature. Since TCNE forms at this temperature multilayer islands before the first monolayer is completed, the IR spectrum of the low-temperature phase at 200 K (blue spectrum in Figure 3b) contains, both, contributions of the monolayer and the condensed multilayer above the monolayer. Comparison with the IR spectrum of crystalline TCNE^46^ allows the sharp, high-intensity bands in the spectrum to be assigned to TCNE multilayer signals. Specifically, the two signals around 2250 cm^–1^ are the IR-active CN stretching modes. The low-wavenumber region (1250–950 cm^–1^) contains the C–C stretching modes at 1255 and 962 cm^–1^ and several combination and overtone bands.^46^ Because of symmetry breaking and interaction with the surface, most of these bands are not observable for the TCNE monolayer species. However, we note the presence of an additional, low-intensity IR signal at around 2175 cm^–1^, which is characteristic for the CN stretching vibration of flat-lying TCNE in the monolayer.^40^ Importantly, a contribution of the symmetric C=C stretching vibration in the 1550–1300 cm^–1^ region is absent in the IR spectrum of the low-temperature phase. While this is expected for the TCNE multilayer for symmetry reasons (this mode is not IR active in TCNE bulk), for molecules at the surface the mode becomes IR active and, considering the metal surface selection rule, should be observed for all adsorbed TCNE molecules, which have the C=C bond, respectively, its transition dipole moment, oriented out of the surface plane. The absence of this signal thus indicates that the low-temperature phase does not contain upright-standing molecules in significant quantities. We note in passing that this does not allow us to completely discard the presence of upright-standing TCNE, since there is an adsorption geometry with the C=C bond parallel to the surface in which the intensity of this vibration theoretically vanishes. As this signal is consistently found for full monolayer coverage at 300 K,^40^ we take this as strong indication that at 200 K the surface consists (almost) exclusively of flat-lying molecules. To test this hypothesis and provide a driving force out of the kinetic trapped state, we subsequently allowed the sample to reach room temperature. During the thawing of the sample, the multilayer desorbs, and all multilayer-related IR signals disappear (red spectrum in Figure 3b). The remaining CN stretching vibrations below and above 2180 cm^–1^ can be assigned to surface-bound and free CN groups of adsorbed TCNE molecules. Significantly, the spectrum does now also contain the characteristic C=C stretching vibration of upright-standing TCNE in contact with the surface at 1368 cm^–1^.^40^ This indicates that a phase transition toward the thermodynamically stable phase has partially taken place. We note that qualitatively, despite all the simplifications, our kMC simulations also suggest the presence of a partially mixed phase near these conditions (see Figure 2).

To understand why in some growth conditions, thermodynamic equilibrium is reached quickly, while in others not, we analyze the simulation of the growth process and its time evolution in more detail. For most conditions, the growth process occurs in two stages. In the initial phase of the growth, every molecule that adsorbs upright-standing can diffuse on the surface and find a free spot to “fall over” to minimize its energy. The reverse process, molecules standing up, is comparatively slow and quickly undone by the same molecule falling over again. Thus, in the first stage of the growth process exclusively flat-lying molecules occupy the surface since this is the phase minimizing the total free energy of the system.

Once the surface is completely covered with molecules, the second stage of the growth process starts. This is characterized by the joint process of a flat-lying molecule standing up and another molecule adsorbing next to it before it falls over again. For realistic (i.e., not too high) pressures and (not too low) temperatures, the rate of falling over is larger than that of adsorption, and hence, the probability for this joint process is relatively small compared to “fluctuating”, i.e., standing up and falling over without adsorption of an additional molecule. As will become important later, we find, for essentially all deposition conditions, that the limiting factor in this joint process is the rate of adsorption. This is the case even at very high pressures and is simply a consequence of the fact that the barrier for the molecule to fall over is very small.

Although adsorption of a standing molecule in the second stage is a rare process, once it does occur, it is hardly undone. None of the two standing molecules can fall over, since the adjacent sites are already occupied with other molecules. The only pathway toward lying molecules would now be to desorb one of the upright-standing molecules. The process of standing up and concurrent adsorption depends both on temperature (for the first part) and pressure (for the second part), while desorption depends solely on temperature (and the adsorption energy of the standing molecules).

Interestingly, the two stages of growth observed here are reminiscent of Ostwald’s rule of stages,^47^ which states that the phase most closely resembling the “mother phase” forms first before the thermodynamically stable phase forms. Although technically a “mother phase” here does not exist, we find that here the phase with the lowest density, which also corresponds to the lowest energy per molecule, inevitably forms first before a thermodynamically more stable phase is formed. We note that, qualitatively, the growth behavior and the formation of a lying phase before a standing phase is the same (without intermolecular interactions) as in self-assembled monolayers (with weak molecule–substrate interactions).^48^

**Figure 4:**
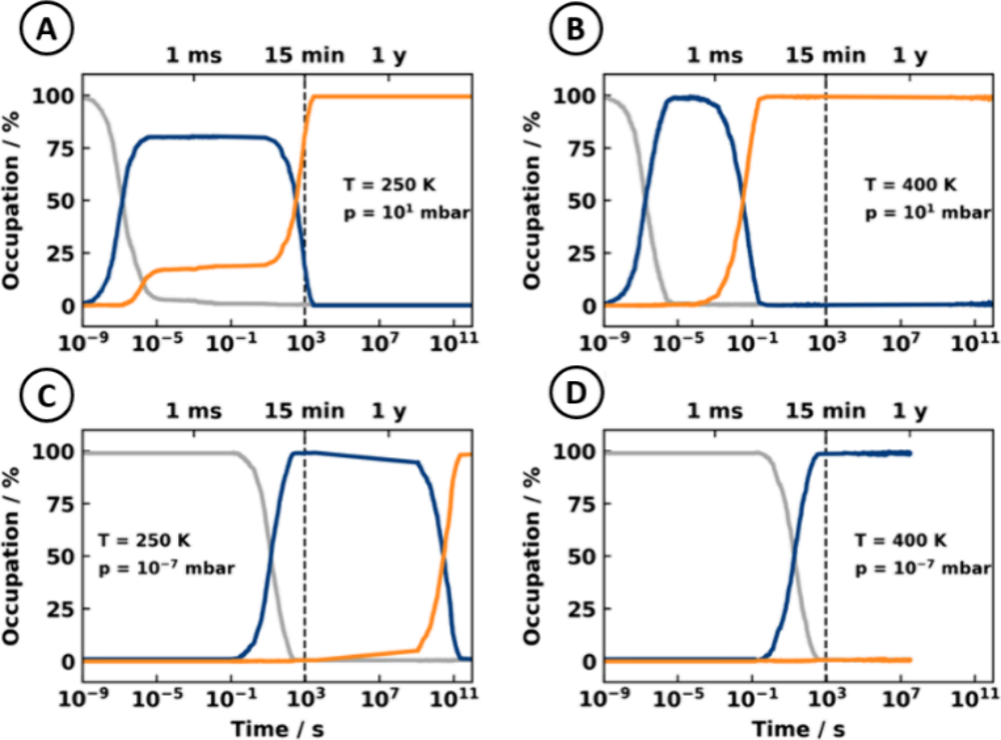
(A–D) Evolutions of the surface composition during Monte Carlo simulations under different conditions highlighted in Figure 2 (see inset); traces show the fraction of surface sites being empty (gray trace), occupied by flat-lying molecules (blue), and occupied by molecules in an upright geometry (orange).

The different dependence of the processes on the pressure and temperature allows us to rationalize the observed behavior after 15 min. Figure 4a–d tracks the surface composition as a function of time for different deposition conditions. As a joint feature in all these plots, we find that initially, the flat-lying phase forms. If both the temperature and deposition rate are high (shown in Figure 4b), the lying molecules often attempt to stand up. At these high background pressures, the high availability of molecules in the gas phase leads to the adsorption of upright-standing molecules. This leads to a very quick phase transition to the thermodynamically stable standing phase in a matter of seconds. Qualitatively, the situation remains similar at lower temperatures (250 K; shown in Figure 4a). Here, molecules standing up occurs less frequently, but again, once they do, another standing molecule is (irreversibly) adsorbed. Due to the lower temperature, it takes approximately 1 h until the phase transition is completed. It is important to note, however, that the deposition rates assumed in Figure 2b and 2a are very high. At 250 K and more realistic deposition rates (ca. 0.1 Å/s), the probability of the joint process of standing up and adsorbing a molecule becomes very low, such that the phase transition only occurs after several years (Figure 4c). This effectively leads to the kinetic trapping of the lying phase. We note that similarly, trapping is also predicted when simply considering the adsorption of hard rods.^42^ Finally, when the adsorbate remains at such low deposition rates but goes to higher temperatures (Figure 4d), the reverse process (standing up and desorption of this molecule, leaving a sufficiently large empty space for flat-lying molecules to adsorb) starts to compete with the joint process of standing up and adsorption of another standing molecule. When the former becomes dominant, the transient population of upright-standing molecules becomes zero. This is tantamount to the flat-lying phase being thermodynamically more stable, and hence, no phase transition to an upright-standing phase occurs at all. Note that at even higher temperature, also the flat-lying molecules start to desorb, leading to surfaces that are only partially covered or entirely molecule-free.

**Figure 5:**
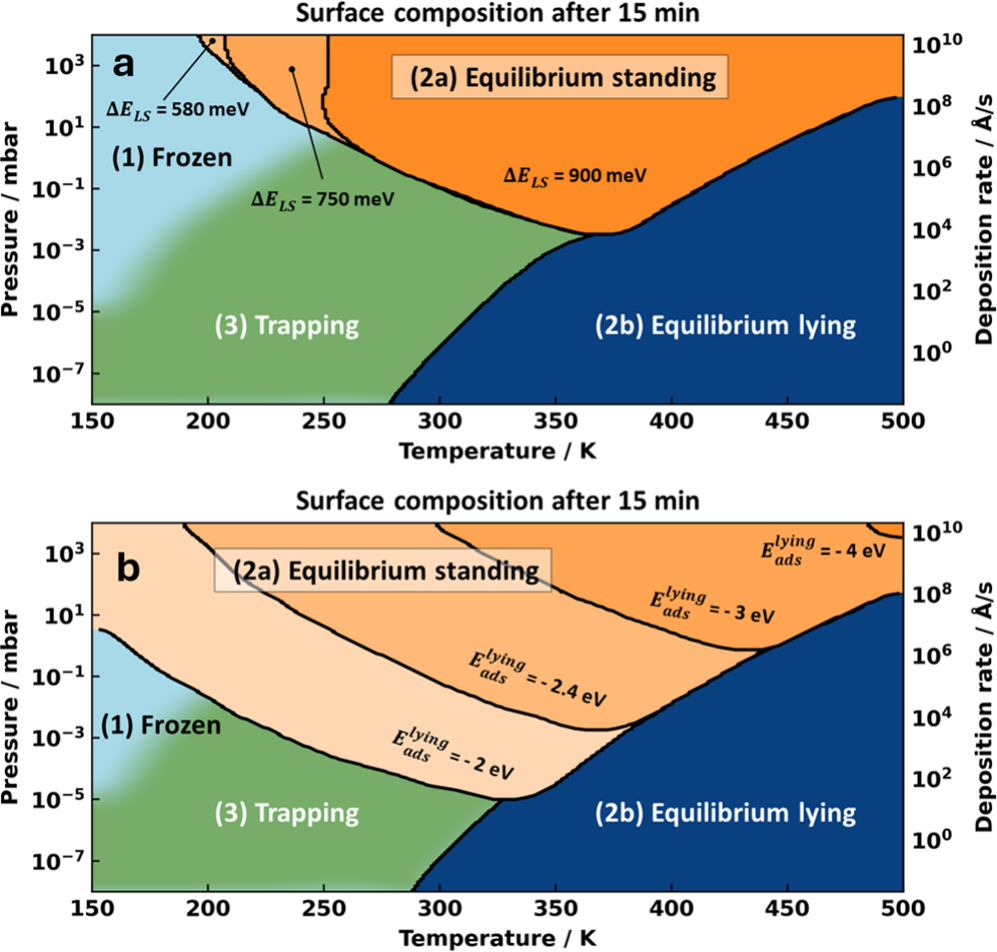
Evolution of the conditions in which thermodynamic equilibrium or kinetic trapping occurs after 15 min of deposition. (a) Varying barriers to stand up and (b) varying adsorption energies with the associated larger barriers to stand up.

An important insight from these considerations is that the deciding factor for the kinetic trapping of the flat-lying phase is mostly the pressure in the gas phase, i.e., the availability of additional molecules. This implies that it is a relatively general phenomenon. In order to test this assumption, we performed additional tests with largely different parameters for the adsorption energies and barriers. As we show in the Supporting Information, increasing the barriers for diffusion has no discernible impact on the growth kinetics at all (see Figure S4). As a second test, we raised the energy of the transition state for the reorientation, i.e., increased the corresponding barrier. (We note in passing that the barrier to fall over is only 40 meV, thus reducing it further is hardly sensible.) As the results shown in Figure 5a demonstrate, this has no effect on the conditions under which kinetic trapping occurs (region 3). Only the onset of the region where thermodynamic equilibrium is already established (2a) is shifted to higher temperatures.

As final test, we varied the adsorption energy of the flat-lying geometry between −2 and −4 eV. To achieve this, we must keep the difference between the adsorption energies per unit area constant. For the sake of comparability to the other situations, the adsorption energy of the upright-standing geometry was adapted such that the phase diagram, i.e., the composition after infinite time, remains unaltered. As Figure 5b shows, going toward larger (more negative) adsorption energies results in a reclining of region 2a, i.e., kinetic trapping of the flat-lying form at the expense of the upright-standing phase becomes more likely. Of course, conversely, smaller (less negative) energies than TCNE’s allow thermodynamic equilibrium to be reached already at smaller deposition rates and lower temperatures. We note, however, that the adsorption energy usually scales (roughly) with the size of the molecule. Since TCNE is already one of the smallest possible conjugated organic molecules, we conclude from these tests that kinetic trapping of the flat-lying phase is very likely for metal–organic interfaces in conditions commonly used in surface science (almost) independently of the nature of the molecule.

## Conclusions

Summarizing, we performed first-principles kinetic Monte Carlo simulations to study the growth of functionalized conjugated organic molecules on metal surfaces. Such molecules are likely to exhibit (at least) two different phases, a flat-lying and an upright-standing geometry. Reminiscent of Ostwald’s rule of stages, we find that growth generally occurs in two stages: First, a low-density phase with the higher adsorption energy per molecule is formed, before eventually the phase transition to the thermodynamically stable phase occurs. For realistic growth conditions, it appears that the limiting factor for the phase transition is the adsorption of additional molecules. Indeed, at low temperatures, we only find experimental indications for flat-lying molecules on the surface, even though a phase consisting of standing molecules should be thermodynamically preferred. Heating the sample to room temperature reduces this kinetic hindering, and under these conditions we do find a mixture of both standing and lying molecules. However, the phase transition does not complete during the time of the experiment. This is consistent with our simulations, which show that the time required to establish thermodynamic equilibrium exceeds several hours even at nominal deposition rates of 100 Å/s.

Interestingly, the obtained results are only weakly dependent on the barriers for diffusion and reorientation. This indicates that the conditions usually employed to grow metal/organic interfaces in surface science experiments (room temperatures and growth rates below 1 Å/s) can readily lead to kinetically trapped phases and may be a reason why upright-standing layers in direct contact with metal surfaces are rarely observed.

## Supporting Information Available

Discussion of the relative thermodynamic stability of upright-standing and flat-lying monolayers on metals; additional methodological details for the kMC simulations, including a more detailed explanation how standing and lying layers were modeled, the values of the barriers chosen; core-level spectroscopy data for TCNE on Cu(111) at different coverages (PDF)
