# Supplementary material for: Kinetic Trapping of Charge-Transfer Molecules at Metal Interfaces
Source: J Phys Chem C Nanomater Interfaces. 2024 Feb 7;128(7):3082–9. doi: 10.1021/acs.jpcc.3c08262 (PMC10895664; doi:10.1021/acs.jpcc.3c08262)
Supplement: Supplementary file 1 — jp3c08262_si_001.pdf [file jp3c08262_si_001.pdf]

Supporting Information for

Kinetic Trapping of Charge-transfer Molecules at Metal Interfaces

Anna Werkovits,[1*] Simon Hollweger, [1*] Max Niederreiter,[2] Thomas Risse,[3] Johannes J. Cartus,[1] Martin Sterrer,[2] Sebastian Matera,[4] and Oliver T. Hofmann[1]

1 Institute of Solid State Physics, Graz University of Technology, Petersgasse 16/II, 8010 Graz, Austria

2 Institute of Physics, University of Graz, Universitätsplatz 5, 8010 Graz, Austria

3 Institut für Chemie und Biochemie, Freie Universität Berlin, Arminallee 22, 14195 Berlin, Germany

4 Theory Department of the Fritz-Haber Institute, Faradayweg 4-6, 14195 Berlin-Dahlem, Germany

*Both authors contributed equally to this work

Inhalt

Thermodynamic Stability 1

kMC representation of adsorption geometries and transition processes 4

Default barriers of kinetic processes 6

Computational details 7

Variation of kMC parameters 7

Core-level spectroscopy data 10

References 11

Thermodynamic Stability

Although most organic/metal interfaces display a flat-lying first layer, it is useful to briefly consider the relative stabilities of flat-lying viz-a-viz upright-standing geometries to estimate under which conditions we would expect the latter to be thermodynamically more favorable. Because organic molecules can be rather complex, the discussion here must be simplified. Specifically, here, we consider organic molecules that are typical for applications in organic electronics and consist of a π-conjugated core (shown in Figure S1a-c in blue) and functional groups such as cyano or carbonyl groups at its periphery (shown in Figure S1a-c in orange). We furthermore assume that the organic molecules only interact mostly via van-der-Waals forces with each other, i.e. assume that no strong, directional bonds (e.g., mediated by surface ad-atoms or through hydrogen bonds) exist.

Following (ab-initio) thermodynamics,[1] the stable structure is the one which minimizes the Gibbs energy per area γ

$$\gamma = \frac{n}{A}\left(\frac{\Delta E}{n} - \mu(T,p)\right) \tag{1}$$

where *n/A* is the number of molecules per area, *ΔE/n* the formation energy per molecule, and μ the chemical potential of the molecular reservoir, which increases with increasing pressure and decreases with increasing temperature. Generally speaking, equation 1 implies that at low chemical potentials, the structure with the best energy per molecule is thermodynamically stable, while higher chemical potentials provide a driving force towards more densely packed structures.

To discuss the energetic competition between flat-lying and upright standing layers, it is useful to conceptually separate ΔE into the contributions arising from molecule-substrate interactions and from molecule interactions, respectively. When the molecule is oriented with the π-plane parallel to the surface, i.e., lies face-on as shown in Figure S1a, all atoms of the molecule are in direct contact with the surface and interact via van-der-Waals forces (indicated as light blue arrows). As a rule of thumb, the typical magnitude of van-der-Waals forces between metals and organic molecules is about 100 meV per atom.[2] Furthermore, functional groups can also create a (partially) covalent bond.[3] Typical values for covalent bonds on surfaces range between 0.5 and 2 eV, and many molecules have multiple functional groups that are in contact at the same time. In addition, also the molecules within the layer interact with each other. Flat-lying molecules have a relatively small contact area to each other, and in the absence of strong directional interactions (adatom-mediated or hydrogen bond), typical interaction energies for molecules like TCNE,[4] acenequinones[5] or anthracene-derivates[6] are ca. 100 meV per molecule. Since this is very small compared to the molecular-substrate interaction, for the purpose of this discussion we consider the molecule-molecule interaction in flat-lying layers negligible. Summarizing the considerations, for small to mid-sized molecules, ΔE on metals typically are about 2-4 eV per molecule (including, exemplarily, for TCNE on Cu(111)[7] , PTCDA on coinage metals,[2] F4TCNQ on Au(111), [8,9] pyrenetetraraone on Cu(111), and many more).

Conversely, in an upright standing molecule, indicated in Figure S1b, only the atoms of the functional group are in direct contact with the surface. Most other atoms are so far away from the surface that their van-der-Waals interaction is negligible. The main contribution to the molecule-substrate-interaction is, thus, the partially covalent bond the molecule forms with the metal. Hence, because standing molecules typically have less functional groups in contact and due to the smaller van-der-Waals interaction with the surface, isolated standing molecules typically bind less strongly (have less negative ΔE/n) than isolated flat-lying molecules. Typical values are between 1-2 eV (e.g. for TCNE on Cu(111),[4] pyrenetetraone on Cu(111), or biphyenylthiole on Au(111)[10,11]). Furthermore, van-der-Waals

interactions between two organic molecules are much smaller than between a molecule and a metal surface, certainly not enough to overpower the $\Delta E/n$ of 2-4 eV obtained above for flat-lying structures

Because the van-der-Waals interactions between two organic molecules are smaller than between a molecule and a metal surface, the upright-standing structures will only rarely have a better energy per molecule than the flat-lying structures. Thus, in the limit of low coverage/dosage/chemical potential, usually flat-lying molecules are likely to prevail. This is also consistent with the general expectation that organic molecules in direct contact with metal substrates form a so-called "wetting layer", consisting of flat-lying molecules. However, because upright-standing molecules can pack more densely, they will eventually be lower in γ for sufficiently large μ (see Figure S1d). Thus, if we only consider monolayers (i.e., molecules in direct contact with the surface), a flat-lying to upright-standing phase transition would be expected for almost any conjugated organic molecule (with functional groups).

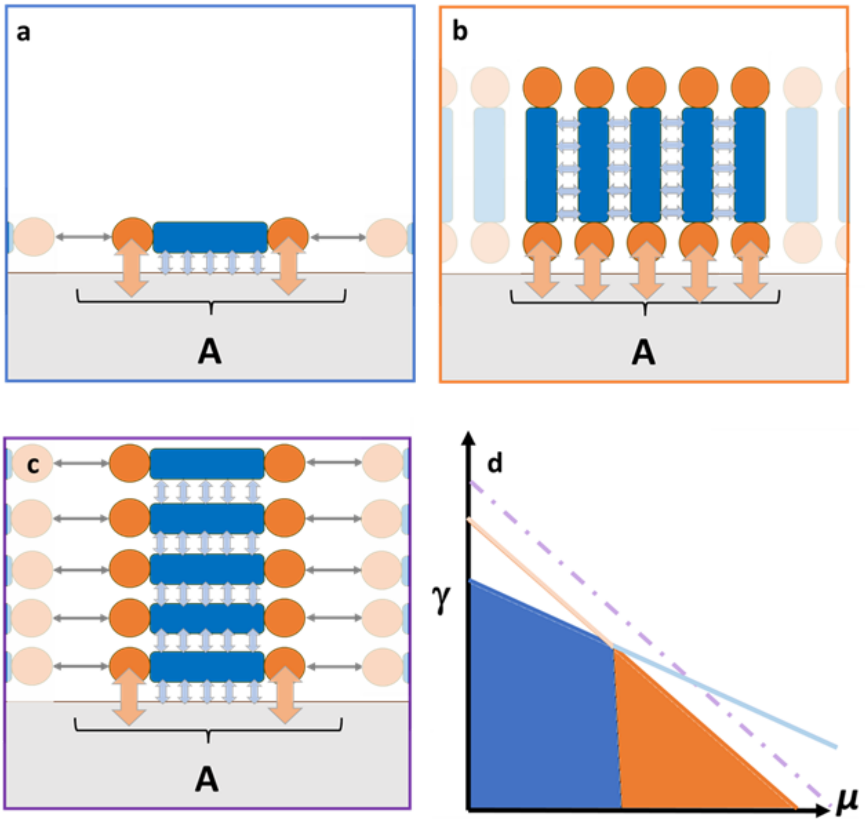

Figure S1: Schematic representations of different structures at interfaces. In (a), a monolayer of flat-lying molecules is shown, where each molecule occupies a certain area A. (b) shows an upright standing geometry. Due to the aspect ration of planar molecules, more molecules can fit into the same area. In (c) a hypothetical multilayer consisting exclusively of flat-lying molecules is shown, which has the same number of molecules per area as the standing geometry in b. In (a) – (c), orange arrows are used to depict interactions of functional groups with the surface, blue arrows denote van-der-Waals interactions, and grey arrows indicate in-plane intermolecular interactions. Panel (d) shows the evolution of the Gibbs free energy per area γ as function of the chemical potential μ. The color of the lines corresponds to color of the boxes around (a)-(c). The energetically most favorable structure is indicated by a filled area.

In practice, to see whether the standing phase indeed becomes stable eventually, it must be compared to a phase with a multilayer structure exhibiting the same number of molecules (schematically shown in Figure S1c). It stands to reason that the interaction between the molecules in the upright-standing monolayer and the flat-lying multilayer, which are both van-der-Waals driven, are similarly large (although, of course, differences in the geometry, the periodicity of the upright-standing monolayer parallel to the surface, and the existence of in-plane interactions in the multilayer may slightly benefit one structure over the other). Conversely, the larger difference stems from the interaction between the molecules and the surface. In a first approximation, we can expect the upright-standing phase to be more stable if the increased density of functional groups in direct contact with the surface outweighs the van-der-Waals interactions of the flat-lying molecule. Since the approximate value for the van-der-Waals interactions is 100 meV/atom (corresponding to ca. 66 meV / Å^2), we can expect a phase transition if additional density of functional groups at the surface for the upright standing layer is so large that this value is exceeded. Depending on the interaction energy of the functional groups (0.5 eV – 2 eV) this should be the case if the area occupied by an upright standing molecule does not exceed ca. 8-30 Å^2 per functional group. Although the discussion presented here is, of course, mostly qualitatively (and works with approximate magnitude of numbers, to which always exceptions can be found), we note that this condition is met for many organic molecules.

kMC representation of adsorption geometries and transition processes

In previous studies 42 different adsorption geometries[7] and 78 on-surface transitions[12] were found in DFT-based simulations of TCNE on Cu(111). These numbers already contain all symmetry equivalents. To make such high throughput kMC simulations of this system possible, the model complexity is tremendously simplified, while the qualitative description of the kinetic effects is retained. As demonstrated in Figure S2, we approximate the adsorption geometries on the originally hexagonal Cu(111) surface by a square lattice with a lattice constant of 3.4 Å. We include only one type of the flat-lying adsorption sites (with a 2x2 footprint covering 11.56 Å^2) and two of the upright-standing ones (with 1x2 and 2x1 footprints, referred to as 'standing vertical' and 'standing horizontal', covering 5.78 Å^2). This representation also approximates the tightest packing found in ref Egger et al.[7] We note here again that intermolecular interactions are not included in this work.

As the adsorption geometries cover several lattice sites, each occupied site is treated separately in the kmcos code (different "species" in the kmcos-jargon). For example, the lying adsorption geometry is represented via the species L0, L1, L2 and L3 (Figure S2c).

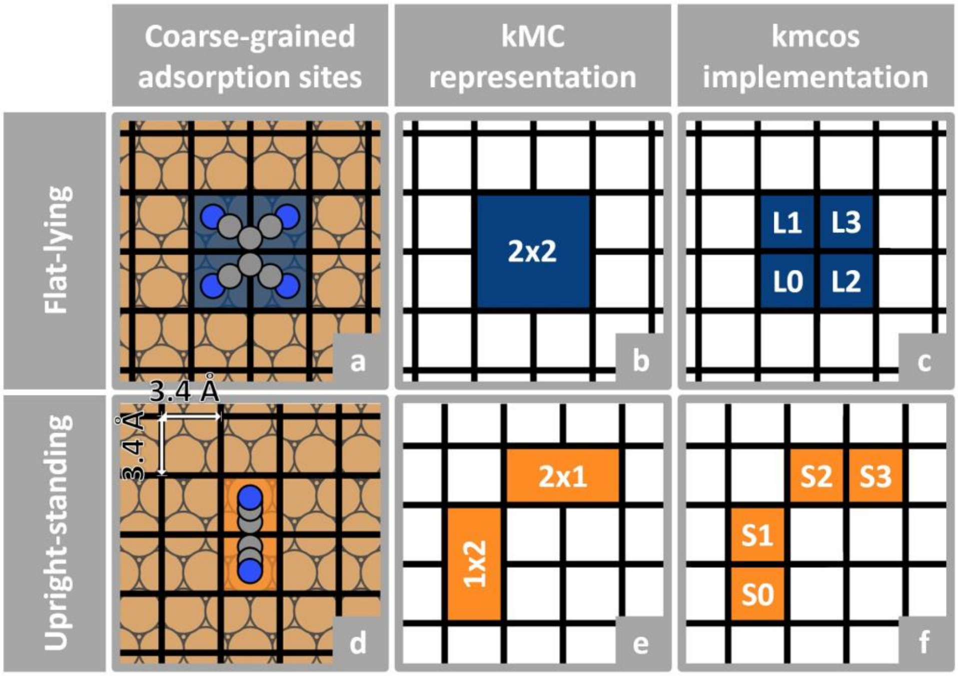

Figure S2: Coarse-graining of DFT-simulated flat-lying (a) and upright-standing (d) adsorption sites for kMC representation in a square lattice with a side length of 3.4 Å. (b) and (e) show the spatial representation, i.e. the footprints, in the kMC representation. (c) and (f) shows how the lying and two standing adsorption geometries are explicitly represented in the kmcos code. Each lattice site, that is occupied by the molecule, is assigned to an own "specie" in the kmcos code to handle the spatial extent of the model when defining transition processes.

Transferring the kinetic processes found in ref[12] to the course-grained adsorption geometries, we figured out 24 on-surface processes that are implemented kMC model. In Figure S3, these are assorted in the groups lying translation, standing translation, standing rotation and standing-up and lying-down. The standing rotation only can take place if the lattice site between the horizontal and vertical orientation is unoccupied. In addition, we added for each adsorption geometry an adsorption and a desorption process – these are in total 6 sorption processes.

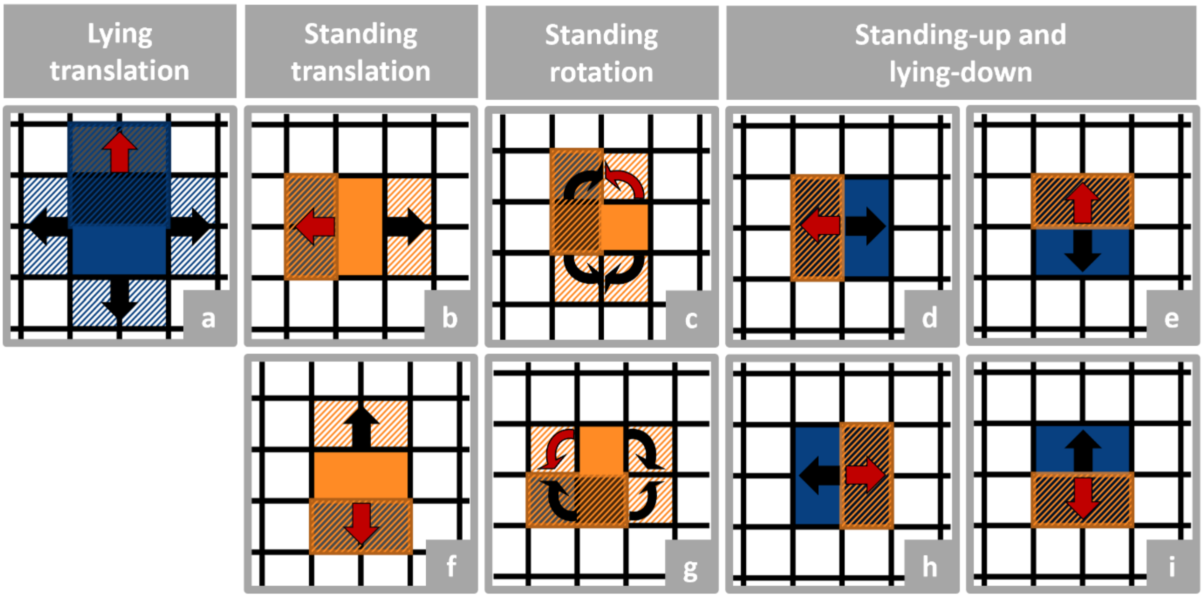

Figure S3: Overview of all on-surface processes implemented in the kMC model. The initial adsorption geometries are displayed as in Figure S2, as filled rectangles. The final geometries, i.e., adsorption sites after the transition processes, are shown in a hatched style, whereas the arrows guide towards the transition direction. As symmetry equivalents (or forward and reverse transitions) are shown together in the single subfigures, in each subfigure one distinct process is highlighted by a red arrow and its corresponding final geometry (darker shade & colored edge). Processes: (a) 4 lying transitions, (b) 2 translations of standing vertical, (f) 2 transitions of standing horizontal, (c)&(g) 8 rotations between standing horizontal and vertical, (d)-(e)&(h)-(f) 8 reorientation processes from lying to standing and vice versa.

Default barriers of kinetic processes

The rate constants of the kMC simulations are all modelled via the Arrhenius equation:

$$k = A \times \exp\left(-\frac{\Delta E}{k_B T}\right)$$

Where k is the rate constant, A the attempt frequency of the corresponding process, and ΔE the energy that must be overcome. Most of the simulations presented in the main manuscript are based on the values of TCNE/Cu(111), which we reported in an earlier paper.[12] As the number of adsorption geometries and correspondingly the number of kinetic processes is reduced, the smallest barriers (ΔE) are chosen for the streamlined process. For the sake of self-containment, we also report them here:

Table S1: Parameters of on-surface and sorption processes. The corresponding on-surface process are displayed in Figure S3. A states the attempt frequency and ΔE the energy barrier of the Arrhenius equation above. For the desorption processes, see description below.

Process	A [s^{-1}]	ΔE [eV]
**Diffusion of lying molecules**	3×10^{14}	0.45
**Diffusion of standing molecules**	5×10^{12}	0.05
**Standing up (lying → standing)**	5×10^{13}	0.58
**Falling over (standing →lying)**	9×10^{11}	0.04
**Ad-/Desorption lying molecules**	Impingement factor (eq. 2)	2.40 [barrierless]
**Ad-/Desorption standing molecules**	Impingement factor (eq. 2)	1.86 [barrierless]

Note that we model the adsorption simply via the impingement factor (equation 2 in the main text), assuming a sticking coefficient of unity and a non-activated process. Since the adsorption is generally much faster than all other steps, modelling it as non-activated does not result in a loss of accuracy. The attempt frequency for the desorption is scaled by the impingement factor to account for detailed balance, as is commonly done in literature for this process.[13]

Computational details

All the kinetic Monte Carlo simulations were performed within the kmcos simulation package.[14] The simulation was performed on a 20x20 square lattice with a side length of 3.4 Å. A time acceleration scheme (*Variable Step Size Method* [15–17]) was used to reach reasonable times at the end of the simulation. The values for the adjustable time acceleration parameters that were used in all simulation runs can be found in Table S2.

Table S2: Parameters used for the time acceleration parameters. The parameter names are the ones used within the kmcos code.

Parameter name	Parameter value
**Buffer_parameter**	100
**Sampling_steps**	20
**Execution_steps**	200
**Threshold_parameter**	0.3

For the adsorption rate we used equation 2 from the main text. This gives the total impingement rate on an area *A*. Given that we are dealing with three distinct adsorption geometries, namely ’lying’, ‘standing vertical’, and ‘standing horizontal’, as previously explained, it was necessary to adjust the individual adsorption rates for these three configurations to collectively match the total adsorption rate derived from equation 2. We did this by assuming a 50/50 split in standing and lying adsorptions. To achieve this adjustment, we assumed an equal 50/50 distribution between standing and lying adsorptions. Consequently, we scaled the lying adsorption rate by a factor of 0.5, while the two standing adsorption rates were scaled by a factor of 0.25 each.

Variation of kMC parameters

a) Variation of adsorption energies

In order to test the general validity of our statements, we varied the adsorption energies of the flat-lying versus the upright-standing molecule such that the surface composition in thermodynamic equilibrium, i.e. at “infinite” time, remains constant. During these variations, we kept the energy barrier to fall over constant at 0.04 eV, which implies that the barrier to stand up increases accordingly. The results are summarized in Figure S3 and discussed in the main text of the manuscript.

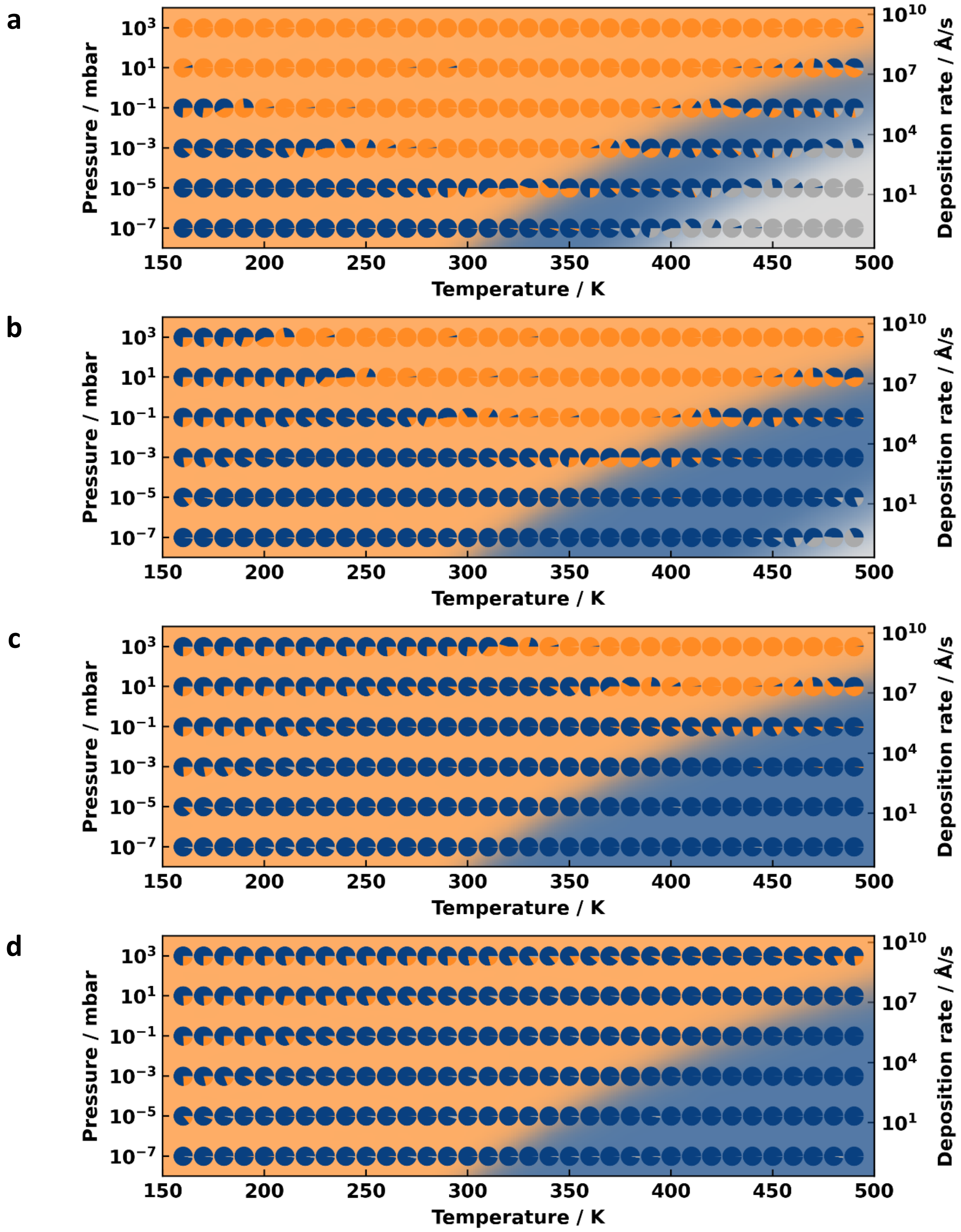

Figure	Adsorption Energy lying molecules	Adsorption Energy standing molecules	Standing up barrier (lying → standing)	Falling over barrier (standing → lying)
a	2.00 eV	1.66 eV	0.38 eV	0.04 eV
b	2.40 eV	1.86 eV	0.58 eV	0.04 eV
c	3.00 eV	2.16 eV	0.88 eV	0.04 eV
d	4.00 eV	2.66 eV	1.38 eV	0.04 eV

Figure S4: The background shows the composition of the surface after infitine time has passed (blue: lying molecules, orange: standing molecules). The the pie-charts represent the relative composition of the surface after 15 min have passed at the same conditions. Blue denotes the percentage of the area covered with lying molecules, orange the percentage of area covered with standing molecules, and grey empty surface. The table below shows the parameters employed in the kMC simulation.

In addition to the barrier variation of the reorientation barrier, we also increased the standing diffusion barrier by 550 meV to a total diffusion barrier of 600 meV. We observed that this change does not have any remarkable impact on the growth kinetics of the standing phase as depicted in Figure S4.

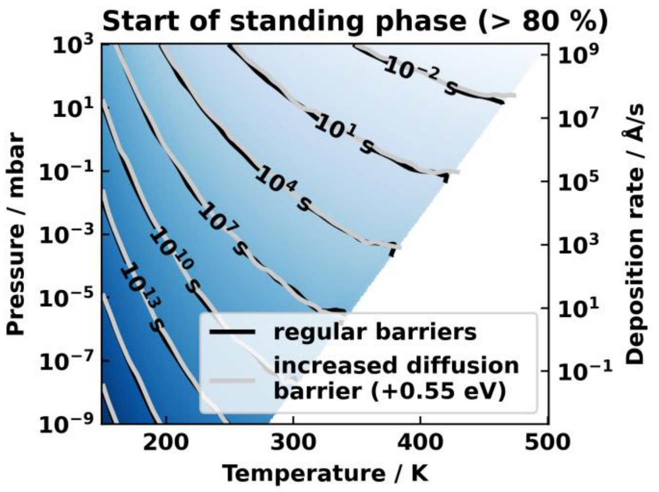

Figure S5: Time to thermodynamic equilibrium of the standing phase, defined by a surface composition of more than 80% standing molecules. Black lines denote the same barriers as defined in the main text, grey lines show the situation for a diffusion barrier increased by 550 meV.

Phase transition times as function of re-orientaiton barrier

An important insight from these considerations is that the limiting factor for the phase transition is mostly the pressure in the gas phase, i.e. availability of additional molecules. This implies that, the relative adsorption energies notwithstanding, it does not depend on the nature of molecule and substrate. In fact, even the height of the barriers should play only a minor role, if any. To test this statement, we repeated the kMC simulations with modified barriers for diffusion on the surface and re-orientation between standing and lying. As we show in the Supporting Information, increasing the barriers for diffusion (by up to 300 meV) has no discernable impact on the growth kinetics (see Figure S4). Maybe more surprisingly, even the barrier governing the re-orientation only has a minor impact for most realistic growth conditions. Figure 5a compares the time until the standing phase is formed for the “regular” barrier for re-orientation (580 meV to stand up, 40 meV to fall over) with a barrier that is increased by 320 meV (i.e., to 900 meV to stand up and 360 meV to fall over). For deposition

rates below ca. 100 Å/s, the time until thermodynamic equilibrium is almost unaffected (within the approximations of the kMC-simulations) by the increased barriers. Only at very low temperatures and/or high deposition rates, the re-orientation becomes the rate-limiting step, i.e. becomes slower than the (re-)adsorption of molecules, as shown in Figure 5b. In this context, it is worthwhile noting that Arefi et al. showed that the re-orientation barriers generally decrease for larger molecules.[18] Since our model molecule TCNE is rather small (10 atoms), we therefore expect that adsorption is the limiting factor for the growth processes of most organic molecules.

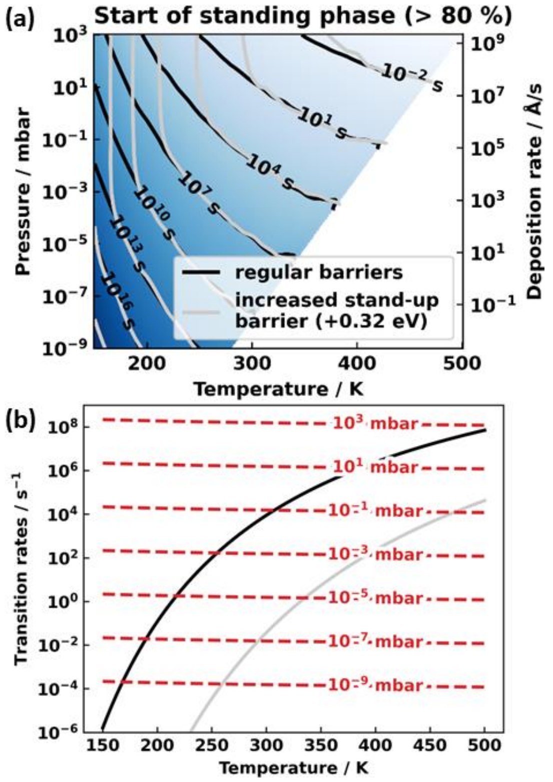

Figure S6: (a) Time to thermodynamic equilibrium of the standing phase, defined by a surface composition of more than 80% standing molecules. Black lines denote the barriers determined for TCNE, grey lines show the situation for a re-orientation 320meV-increased re-orientation barrier. (b) Comparison of impingement rate (red dashed line) at different pressures with the re-orientation rate (standing up) using the barriers for TCNE/Cu (black line) and barriers that are 320 meV larger (grey line).

Core-level spectroscopy data

In Figure S7 we present C 1s (a) and N 1s (b) X-ray photoemission spectroscopy (XPS) spectra of Cu(111) obtained after TCNE saturation doses at room temperature and various pressures (solid lines). In addition, the XP spectrum of the Cu(111) surface with the highest TCNE coverage, which was obtained by depositing a multilayer coverage and subsequent heating to room temperature, is shown for comparison (dashed lines). This coverage is referred to as 1 monolayer (ML) coverage (θ) in this study and corresponds to 3.25 TCNE/nm².

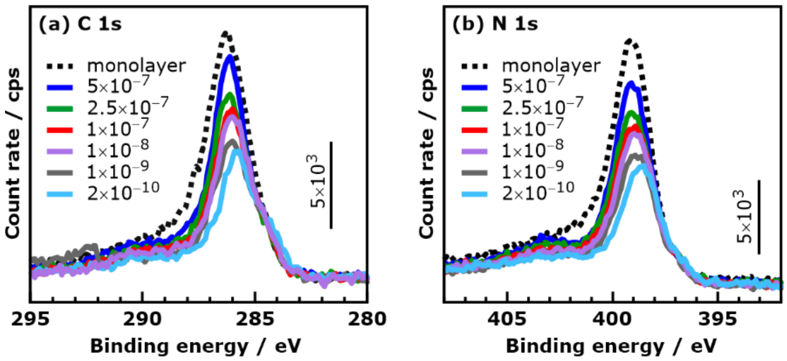

Figure S7: C 1s (a) and N 1s (b) XP spectra for various saturation doses of TCNE at room temperature.

The distribution of lying and standing TCNE molecules on the Cu(111) surface was obtained from fits of the C 1s spectra using fitting parameters reported elsewhere[19]. In short, because of different charge transfer and core-hole screening for lying and standing TCNE molecules, their C 1s (and N 1s) signals are subject to specific binding energy shifts. Examples of XP spectra fits for two different TCNE coverages are presented in Figure S8, where the green and blue fit components represent the different C 1s contributions of lying and standing molecules, respectively (the peaks colored in grey are shake-up satellites). Note the increase of the signals related to standing molecules upon increasing the coverage from 1.65 TCNE/nm² (a) to 2.6 TCNE/nm² (b).

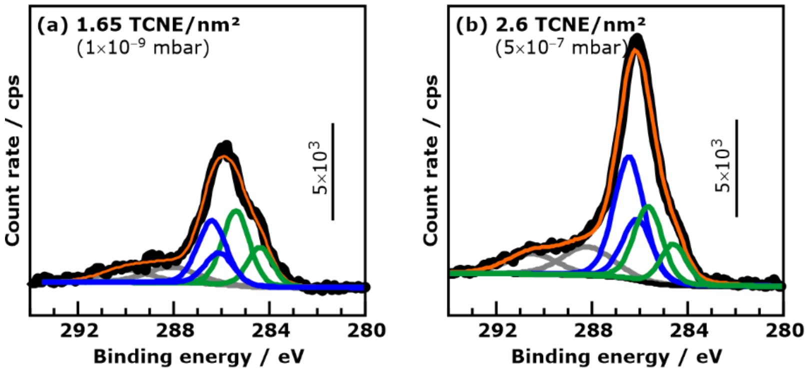

Figure S8: C 1s XP spectra and fit results for saturation TCNE doses at (a) 1x10^{-9} mbar (θ = 1.65 TCNE/nm²) and (b) 5x10^{-7} mbar (θ = 2.6 TCNE/nm²). Black: raw data; green: lying TCNE; blue: standing TCNE; grey: shake-up satellites; orange: sum of fit components.

References

(1) Reuter, K.; Scheffler, M. Composition, Structure, and Stability of RuO 2 (110) as a Function of Oxygen Pressure. *Phys. Rev. B* **2001**, *65* (3), 035406..

(2) Maurer, R. J.; Ruiz, V. G.; Camarillo-Cisneros, J.; Liu, W.; Ferri, N.; Reuter, K.; Tkatchenko, A. Adsorption Structures and Energetics of Molecules on Metal Surfaces: Bridging Experiment and Theory. *Progress in Surface Science* **2016**, *91* (2), 72–100.

(3) Rangger, G.; Hofmann, O.; Romaner, L.; Heimel, G.; Bröker, B.; Blum, R.-P.; Johnson, R.; Koch, N.; Zojer, E. F4TCNQ on Cu, Ag, and Au as Prototypical Example for a Strong Organic Acceptor on Coinage Metals. *Phys. Rev. B* **2009**, *79* (16), 165306.

(4) Cartus, J. J.; Jeindl, A.; Werkovits, A.; Hörmann, L.; Hofmann, O. T. Polymorphism Mediated by Electric Fields: A First Principles Study on Organic/Inorganic Interfaces. *Nanoscale Adv.* **2023**, *5* (8), 2288–2298.

(5) Jeindl, A.; Domke, J.; Hörmann, L.; Sojka, F.; Forker, R.; Fritz, T.; Hofmann, O. T. Nonintuitive Surface Self-Assembly of Functionalized Molecules on Ag(111). *ACS Nano* **2021**, acsnano.0c10065.
(6) Packwood, D. M.; Han, P.; Hitosugi, T. Chemical and Entropic Control on the Molecular Self-Assembly Process. *Nat Commun* **2017**, *8* (1), 14463.
(7) Egger, A. T.; Hörmann, L.; Jeindl, A.; Scherbela, M.; Obersteiner, V.; Todorović, M.; Rinke, P.; Hofmann, O. T. Charge Transfer into Organic Thin Films: A Deeper Insight through Machine-Learning-Assisted Structure Search. *Adv. Sci.* **2020**, 2000992.
(8) Berger, R. K.; Jeindl, A.; Hörmann, L.; Hofmann, O. T. Role of Adatoms for the Adsorption of F4TCNQ on Au(111). *J. Phys. Chem. C* **2022**, *126* (17), 7718–7727.
(9) Blowey, P. J.; Sohail, B.; Rochford, L. A.; Lafosse, T.; Duncan, D. A.; Ryan, P. T. P.; Warr, D. A.; Lee, T.-L.; Costantini, G.; Maurer, R. J.; Woodruff, D. P. Alkali Doping Leads to Charge-Transfer Salt Formation in a Two-Dimensional Metal–Organic Framework. *ACS Nano* **2020**, acsnano.0c03133.
(10) Heimel, G.; Romaner, L.; Bredas, J. L.; Zojer, E. Organic/Metal Interfaces in Self-Assembled Monolayers of Conjugated Thiols: A First-Principles Benchmark Study. *Surface Science* **2006**, *600* (19), 4548–4562.
(11) Bilić, A.; Reimers, J. R.; Hush, N. S. The Structure, Energetics, and Nature of the Chemical Bonding of Phenylthiol Adsorbed on the Au(111) Surface: Implications for Density-Functional Calculations of Molecular-Electronic Conduction. *The Journal of Chemical Physics* **2005**, *122* (9), 094708.
(12) Werkovits, A.; Jeindl, A.; Hörmann, L.; Cartus, J. J.; Hofmann, O. T. Toward Targeted Kinetic Trapping of Organic–Inorganic Interfaces: A Computational Case Study. *ACS Phys. Chem Au* **2022**, *2* (1), 38–46.
(13) Andersen, M.; Plaisance, C. P.; Reuter, K. Assessment of Mean-Field Microkinetic Models for CO Methanation on Stepped Metal Surfaces Using Accelerated Kinetic Monte Carlo. *The Journal of Chemical Physics* **2017**, *147* (15), 152705.
(14) Hoffmann, M. J.; Matera, S.; Reuter, K. Kmos: A Lattice Kinetic Monte Carlo Framework. *Computer Physics Communications* **2014**, *185* (7), 2138–2150. h
(15) Bortz, A. B.; Kalos, M. H.; Lebowitz, J. L. A New Algorithm for Monte Carlo Simulation of Ising Spin Systems. *Journal of Computational Physics* **1975**, *17* (1), 10–18.
(16) Jansen, A. P. J. Monte Carlo Simulations of Chemical Reactions on a Surface with Time-Dependent Reaction-Rate Constants. *Computer Physics Communications* **1995**, *86* (1–2), 1–12.
(17) Gillespie, D. T. A General Method for Numerically Simulating the Stochastic Time Evolution of Coupled Chemical Reactions. *Journal of Computational Physics* **1976**, *22* (4), 403–434.
(18) Arefi, H. H.; Corken, D.; Tautz, F. S.; Maurer, R. J.; Wagner, C. Design Principles for Metastable Standing Molecules. *J. Phys. Chem. C* **2022**, *126* (15), 6880–6891.
(19) Lach, S.; Altenhof, A.; Shi, S.; Fahlman, M.; Ziegler, C. Electronic and Magnetic Properties of a Ferromagnetic Cobalt Surface by Adsorbing Ultrathin Films of Tetracyanoethylene. *Phys. Chem. Chem. Phys.* **2019**, *21* (28), 15833–15844.
